# Supplementary material for: Study of changes in brain dynamics during sleep cycles in dogs under effect of trazodone
Source: PLoS One. 2025 Nov 25;20(11):e0335159. doi: 10.1371/journal.pone.0335159 (PMC12646450; doi:10.1371/journal.pone.0335159)
Supplement: S1 Table — The third column reports the percentage of artifact-free (good) epochs in each state relative to the total number of recorded epochs. (PDF) [file pone.0335159.s001.pdf]

Table 1: Mean $\pm$ standard deviation of the number of clean and noisy epochs across all dogs. The third column reports the percentage of artifact-free (good) epochs in each state relative to the total number of recorded epochs.

**State**	**Control**			**Trazodone**		
	Clean	Noisy	% Good	Clean	Noisy	% Good
Wake	66.3 ± 50.7	848.3 ± 303.0	7.7	198.0 ± 143.9	920.9 ± 394.4	17.7
Drowsiness	178.1 ± 86.7	359.7 ± 318.0	41.63	421.8 ± 225.6	294.8 ± 160.8	58.9
REM	50.8 ± 65.6	100.8 ± 122.8	33.5	7.7 ± 13.9	6.8 ± 12.1	53.1
NREM	635.3 ± 287.8	0	100.0	394.0 ± 349.5	0	100.0
