# Supplementary material for: Study of changes in brain dynamics during sleep cycles in dogs under effect of trazodone
Source: PLoS One. 2025 Nov 25;20(11):e0335159. doi: 10.1371/journal.pone.0335159 (PMC12646450; doi:10.1371/journal.pone.0335159)
Supplement: S2 Table — (PDF) [file pone.0335159.s002.pdf]

Table 2: Number of clean and noisy epochs, and the percentage of clean epochs from total epochs per dog analyzed in this study.

Dog	Condition	Wake Clean	Wake Noisy	Wake %	Drow Clean	Drow Noisy	Drow %	REM Clean	REM Noisy	REM %	NREM Clean	REM %
1	Control	67	650	10,31	130	172	75,58	29	61	47,54	1227	100
	Trazodone	162	808	20,05	178	78	228,21	2	2	100	378	100
2	Control	114	1010	11,29	137	177	77,4	13	25	52	722	100
	Trazodone	105	487	21,56	211	334	63,17	0	0	0	834	100
3	Control	23	745	3,09	80	254	31,5	7	18	38,89	578	100
	Trazodone	593	1131	52,43	377	216	174,54	0	0	0	83	100
4	Control	39	833	4,68	342	529	64,65	21	53	39,62	583	100
	Trazodone	87	1244	6,99	238	589	40,41	0	0	0	90	100
5	Control	208	1071	19,42	299	411	72,75	0	0	0	104	100
	Trazodone	156	764	20,42	591	392	150,77	16	16	100	465	100
6	Control	13	649	2	108	1331	8,11	28	24	116,67	247	100
	Trazodone	218	437	49,89	336	54	622,22	35	33	106,06	987	100
7	Control	52	622	8,36	158	340	46,47	121	160	75,62	750	100
	Trazodone	167	714	23,39	924	525	176	0	0	0	70	100
8	Control	34	348	9,77	174	145	120	244	426	57,28	943	100
	Trazodone	119	589	20,2	791	254	311,42	39	31	125,81	577	100
9	Control	81	1585	5,11	118	109	108,26	21	26	80,77	428	100
	Trazodone	177	1475	12	274	411	66,67	0	0	0	63	100
10	Control	82	1113	7,37	71	201	35,32	53	267	19,85	581	100
	Trazodone	129	502	25,7	336	323	104,02	0	0	0	941	100
11	Control	37	700	5,29	234	219	106,85	25	27	92,59	843	100
	Trazodone	68	1627	4,18	494	123	401,63	0	0	0	0	100
12	Control	46	853	5,39	286	428	66,82	47	122	38,52	618	100
	Trazodone	395	1273	31,03	312	238	131,09	0	0	0	240	100
