# Supplementary material for: Study of changes in brain dynamics during sleep cycles in dogs under effect of trazodone
Source: PLoS One. 2025 Nov 25;20(11):e0335159. doi: 10.1371/journal.pone.0335159 (PMC12646450; doi:10.1371/journal.pone.0335159)
Supplement: S3 Table — (PDF) [file pone.0335159.s003.pdf]

Table 3: Time spent (min) in each behavioral state for every dog under control and trazodone conditions

Dog	Wake		Drow		NREM		REM		Total	
	Control	Traz	Control	Traz	Control	Traz	Control	Traz	Control	Traz
1	32,5	40,4	15,1	12,8	61,35	18,9	4,5	0,2	113,45	72,3
2	50,5	24,35	15,7	27,25	36,1	41,7	1,9	0	104,2	93,3
3	37,25	56,55	16,7	29,65	28,9	4,15	1,25	0	84,1	90,35
4	41,65	62,2	43,55	41,35	29,15	4,5	3,7	0	118,05	108,05
5	53,55	38,2	35,5	49,15	5,2	23,25	0	1,6	94,25	112,2
6	32,45	21,85	71,95	19,5	12,35	49,35	2,6	3,4	119,35	94,1
7	31,1	35,7	24,9	72,45	37,5	3,5	14,05	0	107,55	111,65
8	17,4	29,45	15,95	52,25	47,15	28,85	33,5	3,5	114	114,05
9	79,25	73,75	11,35	34,25	21,4	3,15	2,35	0	114,35	111,15
10	55,65	25,1	13,6	32,95	29,05	47,05	16	0	114,3	105,1
11	35	81,35	22,65	30,85	42,15	0	2,6	0	102,4	112,2
12	42,65	63,65	35,7	27,5	30,9	12	8,45	0	117,7	103,15
