# Supplementary material for: Study of changes in brain dynamics during sleep cycles in dogs under effect of trazodone
Source: PLoS One. 2025 Nov 25;20(11):e0335159. doi: 10.1371/journal.pone.0335159 (PMC12646450; doi:10.1371/journal.pone.0335159)
Supplement: S4 Table — (PDF) [file pone.0335159.s004.pdf]

Table 4: Latency to each sleep state (min) for individual dogs under control and trazodone conditions.

Dogs	Drow		NREM		REM	
	Control	Traz	Control	Traz	Control	Traz
1	1,10	2,17	2,50	2,25	8,60	4,85
2	1,70	2,55	3,23	3,48	29,88	32,85
3	1,57	7,73	1,65	13,08	14,63	40,00
4	1,18	1,05	1,32	17,55	6,37	37,47
5	0,38	1,15	3,75	5,83	34,88	12,65
6	0,35	6,23	22,50	12,75	32,23	22,15
7	0,40	1,00	1,10	20,23	1,93	40,00
8	3,18	2,05	4,27	16,00	5,62	32,30
9	9,48	8,40	15,48	26,83	18,48	40,00
10	0,67	1,65	4,67	2,03	7,65	37,18
11	1,17	3,62	8,23	38,53	8,98	38,53
12	1,47	12,27	17,10	26,58	17,38	40,97
