# Supplementary figures and images for: Study of changes in brain dynamics during sleep cycles in dogs under effect of trazodone
Source: PLoS One. 2025 Nov 25;20(11):e0335159. doi: 10.1371/journal.pone.0335159 (PMC12646450; doi:10.1371/journal.pone.0335159)

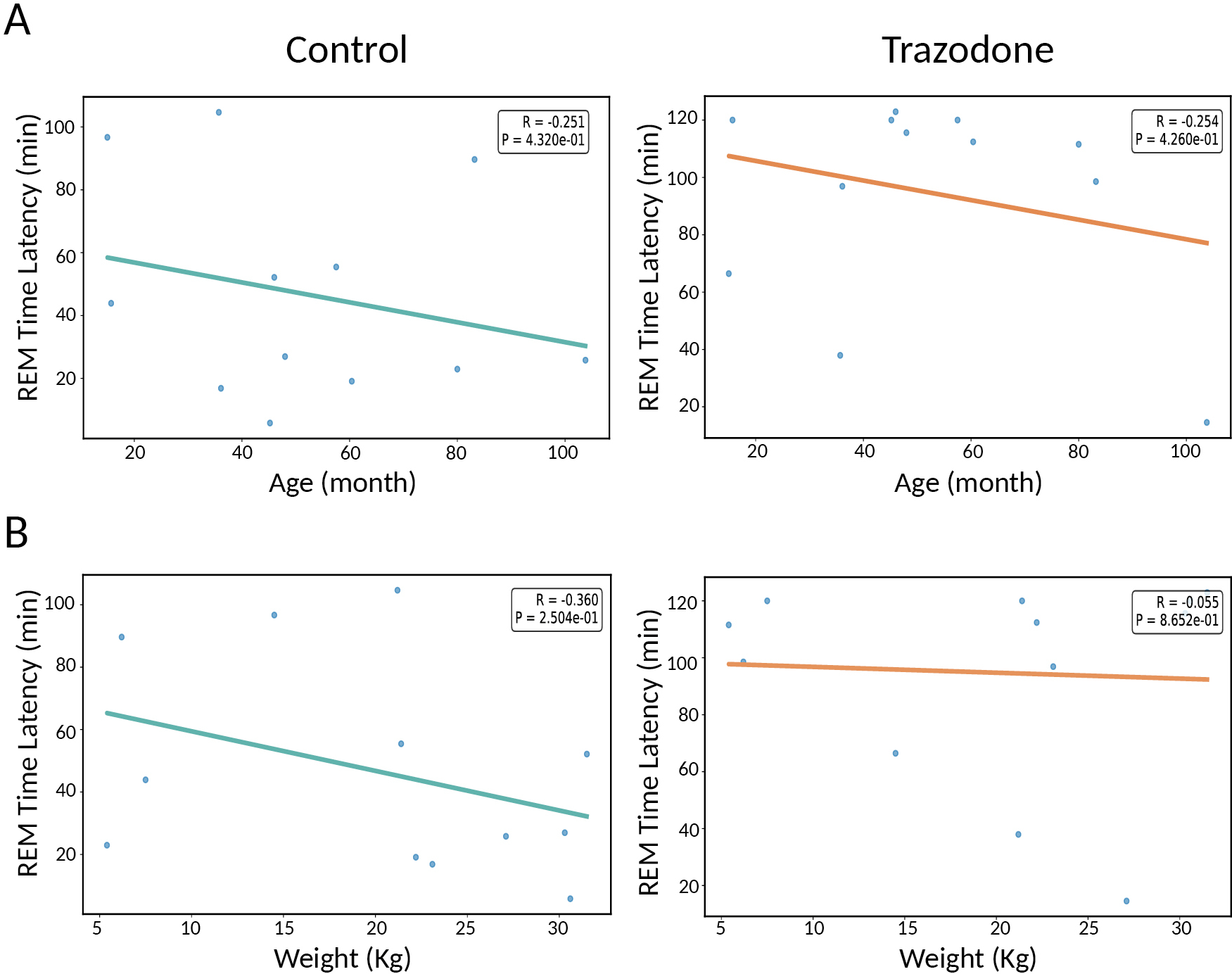

Supplement: S5 Fig — (TIFF) [file pone.0335159.s005.tif]

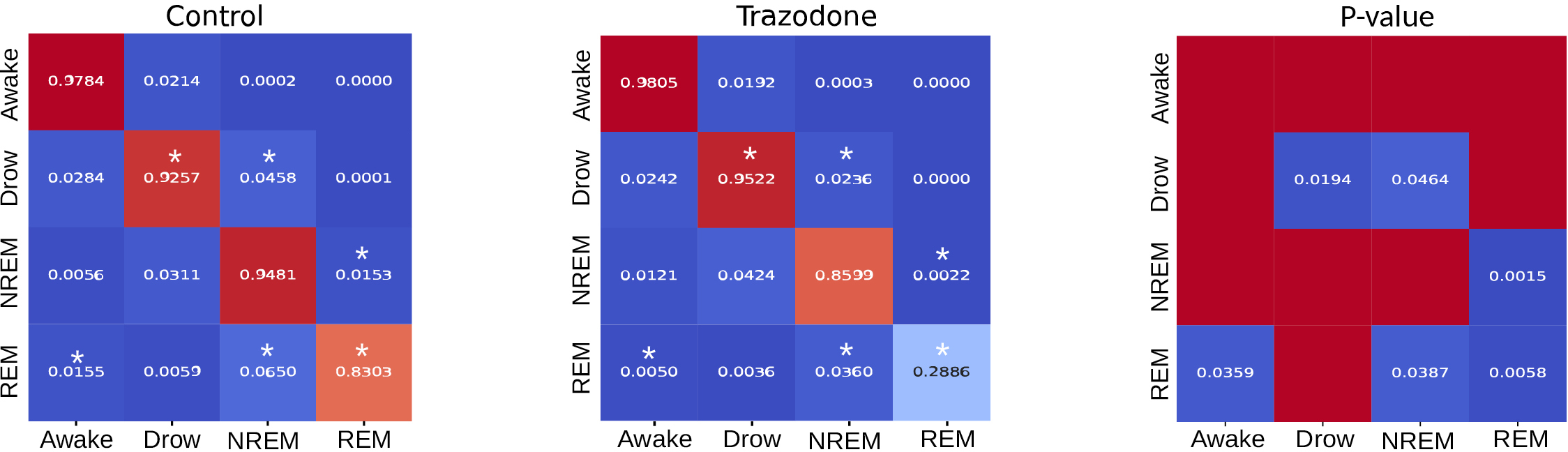

Supplement: S6 Fig — Asterisks indicate statistically significant differences. p-values represent comparisons between matrices in both conditions. Statistical analysis was performed using the Kruskal–Wallis test followed by Dunn’s post hoc correction. (TIFF) [file pone.0335159.s006.tif]

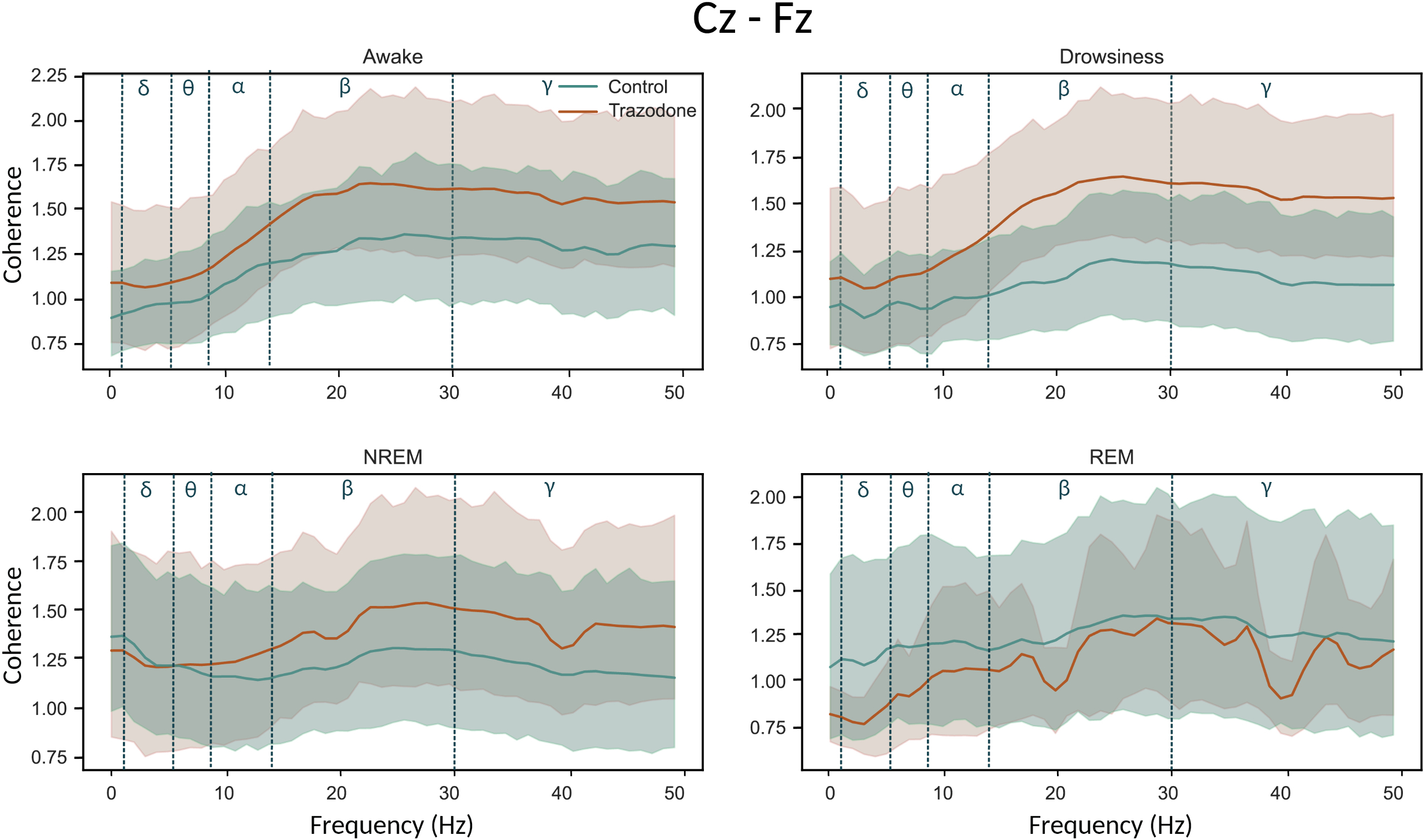

Supplement: S9 Fig — The green and red traces denote the control and trazodone groups, respectively. Vertical blue dashed lines indicate boundaries of standard physiological frequency bands (delta δ, tetha θ, alpha α, beta β, gamma γ). Observed attenuations near 20 Hz and 40 Hz likely reflect harmonic suppression by the notch filter. (TIFF) [file pone.0335159.s009.tif]

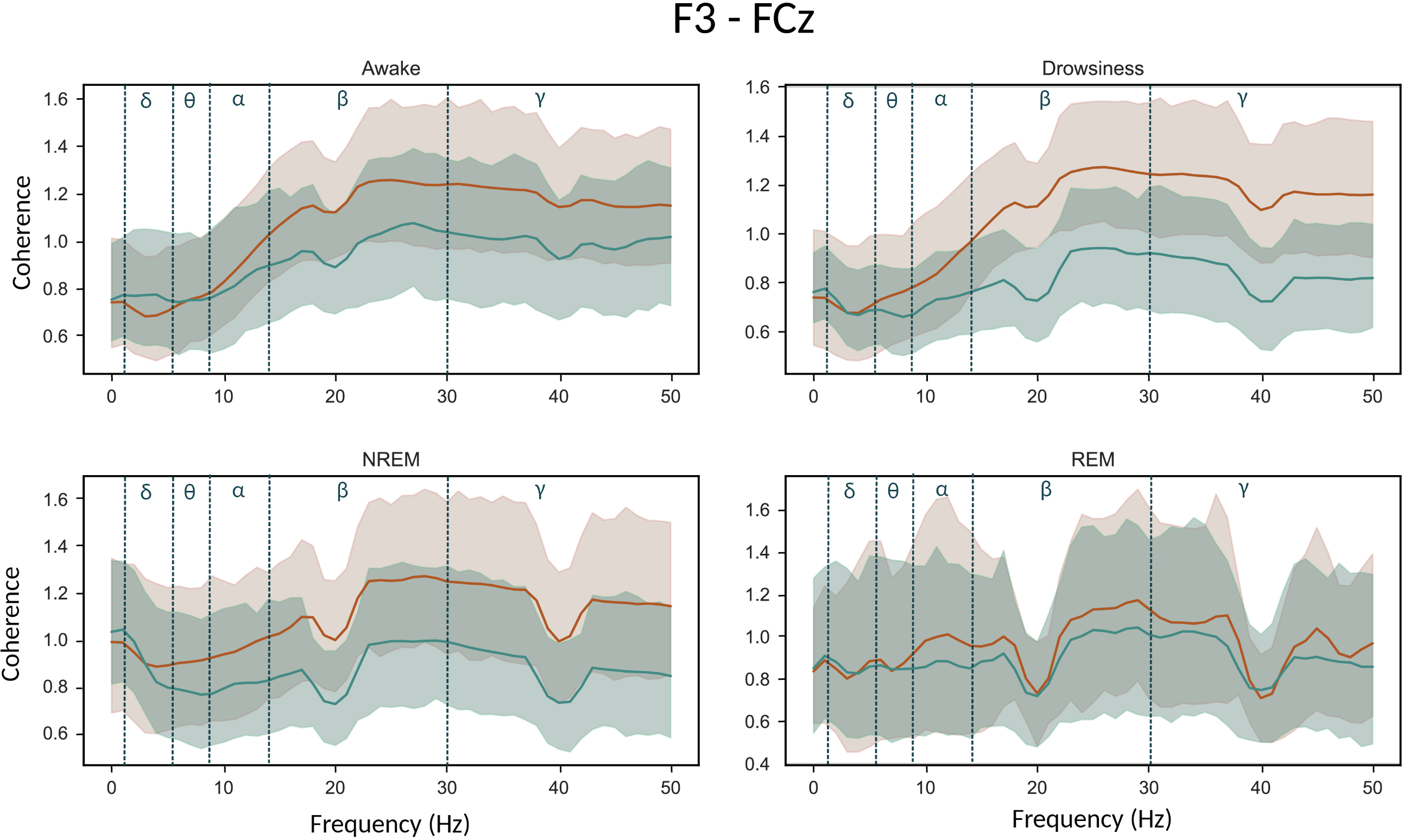

Supplement: S10 Fig — (TIFF) [file pone.0335159.s010.tif]

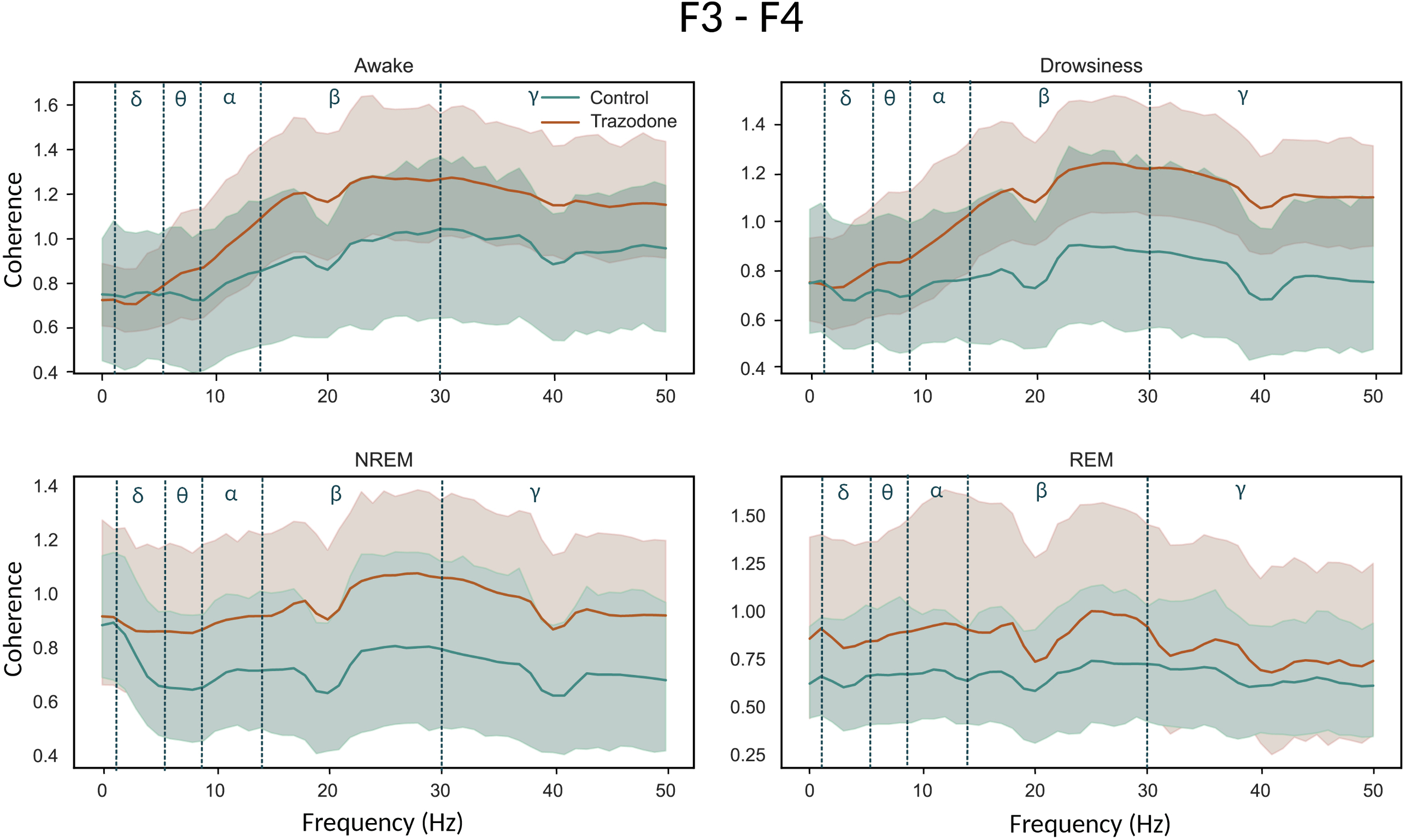

Supplement: S11 Fig — (TIFF) [file pone.0335159.s011.tif]

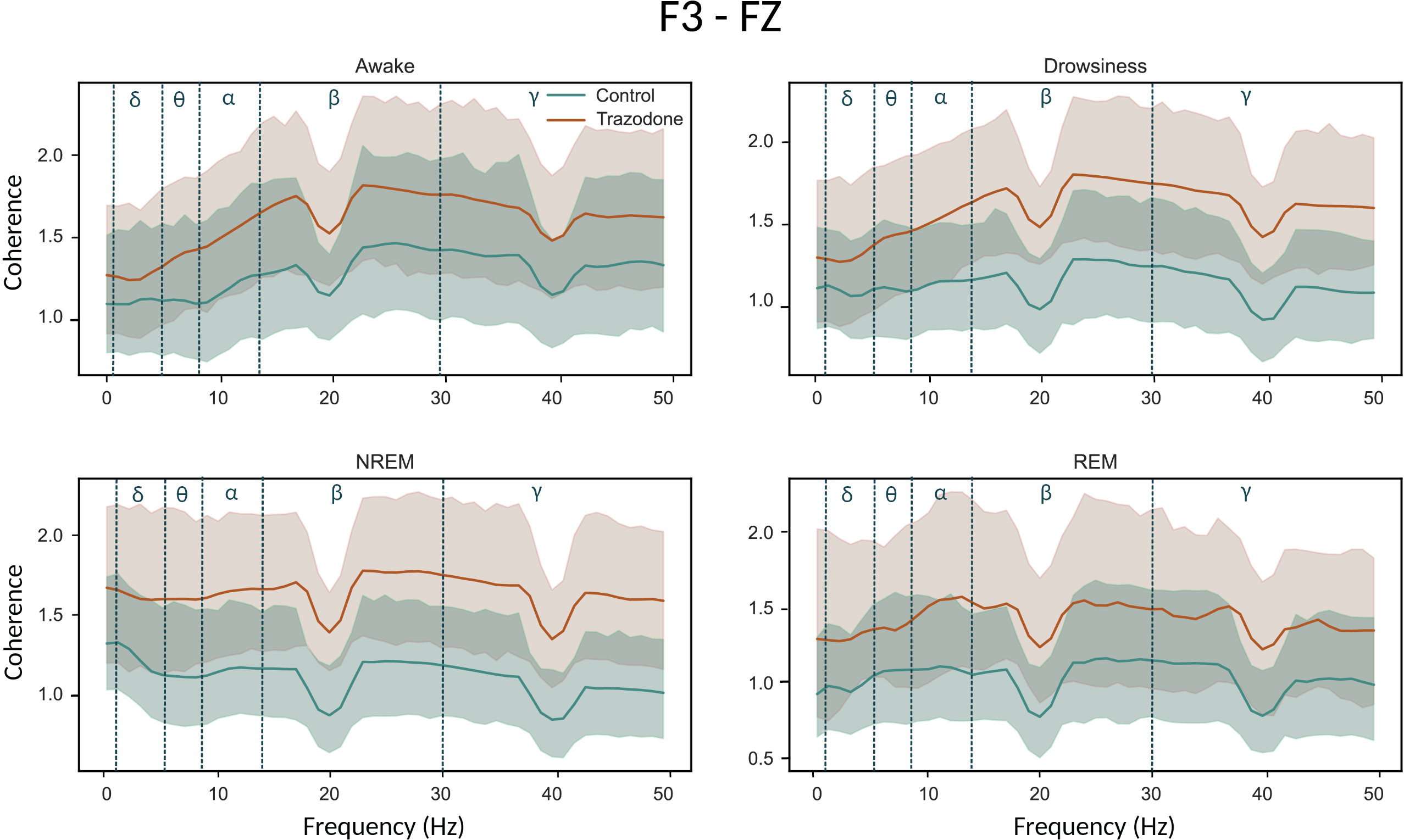

Supplement: S12 Fig — (TIFF) [file pone.0335159.s012.tif]

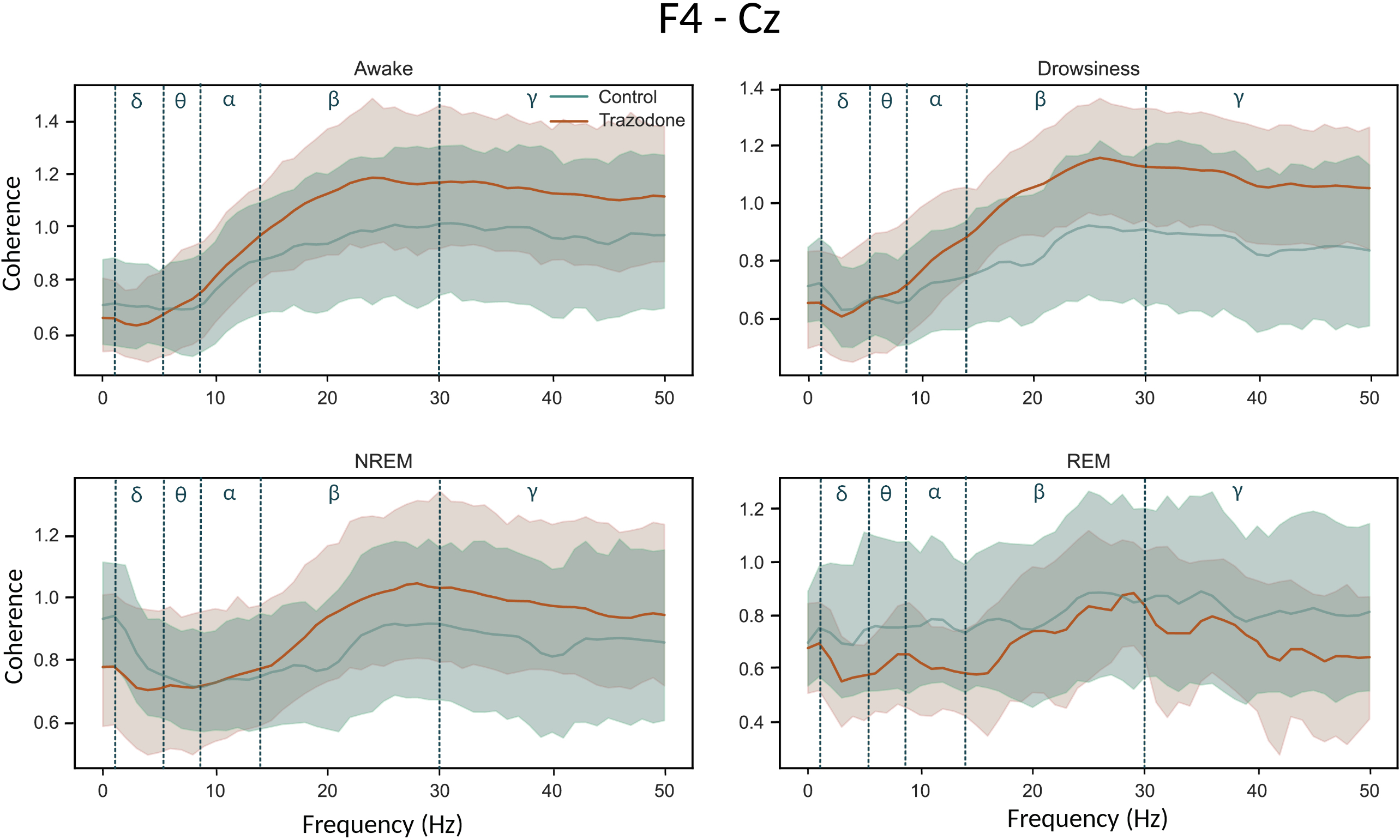

Supplement: S13 Fig — (TIFF) [file pone.0335159.s013.tif]

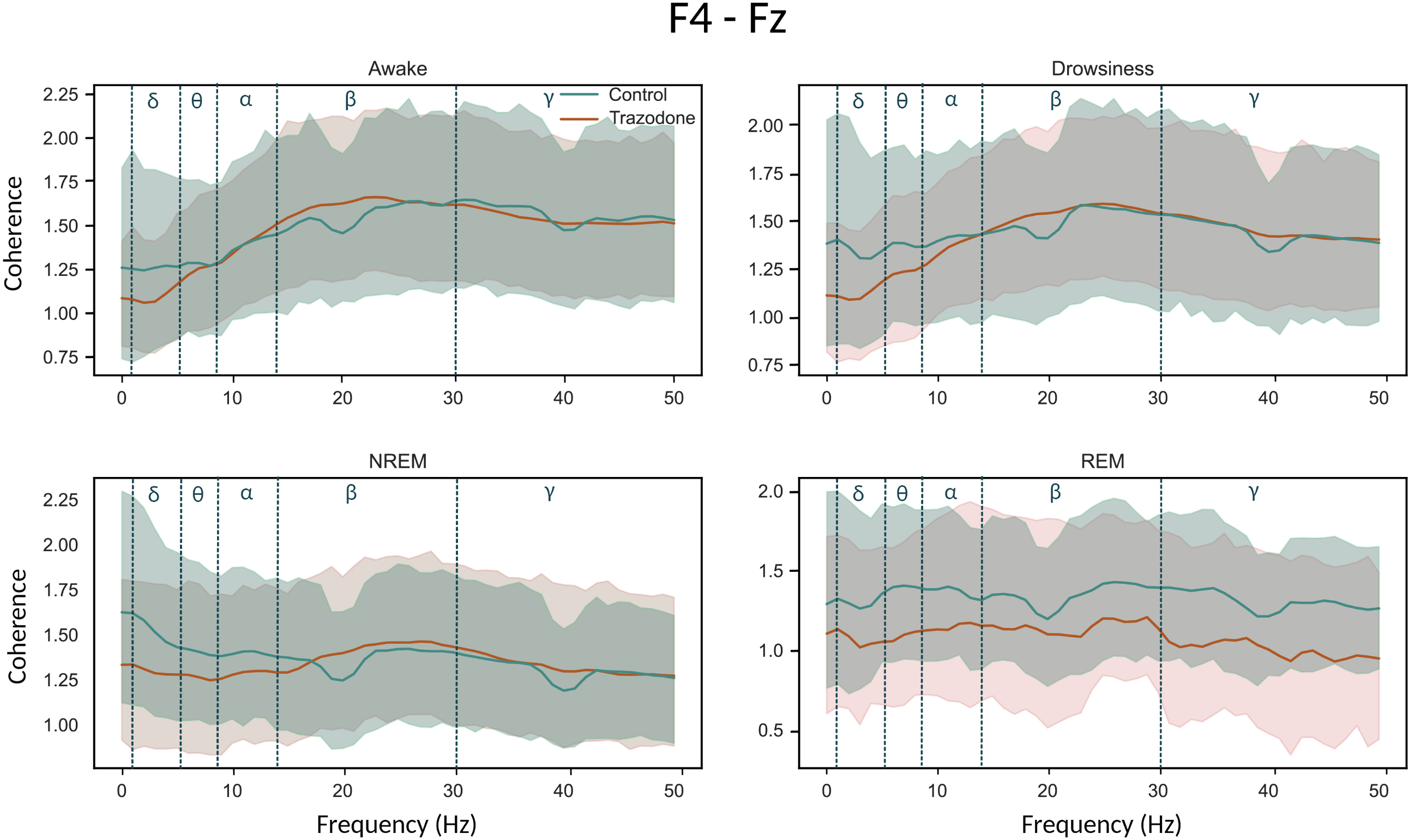

Supplement: S14 Fig — (TIFF) [file pone.0335159.s014.tif]
