# Supplementary material for: Study of changes in brain dynamics during sleep cycles in dogs under effect of trazodone
Source: PLoS One. 2025 Nov 25;20(11):e0335159. doi: 10.1371/journal.pone.0335159 (PMC12646450; doi:10.1371/journal.pone.0335159)
Supplement: S7 Table — p–values were calculated using Wilcoxon signed-rank tests for paired comparisons Wakefulness (Wake) and Drowsiness (Drow), and Wilcoxon rank-sum tests for unpaired comparisons (NREM vs. REM). n/s: not significant (p > 0.05). (PDF) [file pone.0335159.s007.pdf]

Table 5: Statistical significance of Lempel-Ziv Complexity differences in low and high frequency bands between experimental conditions. p–values were calculated using Wilcoxon signed-rank tests for paired comparisons Wakefulness (Wake) and Drowsiness (Drow), and Wilcoxon rank-sum tests for unpaired comparisons (NREM vs. REM). n/s: not significant ($p > 0.05$).

State	Low Frequensy		High Frequnsy	
	Paried	Unpaired	Paried	Unpaired
Wake	0.007	–	n/s	–
Drow	0.002	–	0.001	–
NREM	–	0.003	–	n/s
REM	–	n/s	–	n/s
