# Supplementary material for: Study of changes in brain dynamics during sleep cycles in dogs under effect of trazodone
Source: PLoS One. 2025 Nov 25;20(11):e0335159. doi: 10.1371/journal.pone.0335159 (PMC12646450; doi:10.1371/journal.pone.0335159)
Supplement: S8 Table — p–values were calculated using Wilcoxon signed-rank tests for paired comparisons Wakefulness (Wake) and Drowsiness (Drow), and Wilcoxon rank-sum tests for unpaired comparisons (NREM vs. REM). n/s: not significant (p > 0.05). (PDF) [file pone.0335159.s008.pdf]

Table 6: Statistical significance of Permutation Entropy differences in low and high frequency bands between experimental conditions. p–values were calculated using Wilcoxon signed–rank tests for paired comparisons Wakefulness (Wake) and Drowsiness (Drow), and Wilcoxon rank-sum tests for unpaired comparisons (NREM vs. REM). n/s: not significant ($p > 0.05$).

State	Low Frequensy		High Frequnsy	
	Paried	Unpaired	Paried	Unpaired
Wake	0.005	–	n/s	–
Drow	0.009	–	0.009	–
NREM	–	0.017	–	n/s
REM	–	n/s	–	n/s
