# Supplementary material for: Study of changes in brain dynamics during sleep cycles in dogs under effect of trazodone
Source: PLoS One. 2025 Nov 25;20(11):e0335159. doi: 10.1371/journal.pone.0335159 (PMC12646450; doi:10.1371/journal.pone.0335159)
Supplement: Attachment — Submitted filename: Answer_vf.pdf [file pone.0335159.s015.pdf]

Response to Reviewer

We would like to sincerely thank the reviewer for their careful evaluation of our manuscript and for the constructive comments and suggestions provided.

In the revised version, we have carefully addressed all comments point by point. Below, we provide detailed responses to each remark, including clarifications, additional analyses, and textual revisions where appropriate. All corresponding changes have been incorporated into the manuscript, and newly added or modified texts were written in red in the revised document for clarity.

1- How much dog size (e.g. breed) affect to usual health and physiological function altogether and therefore to your results also? As I have understood smaller breeds live longer than bigger ones, e.g. Bernese mountain dog (~8 years) vs. Tibetan Spaniel (~14 years), as in your dataset there are also bigger (e.g. labrador) and smaller (maltese) breeds?

We acknowledge that different breeds can produce variable recordings. Skull size, for example, can influence electrode placement and the specific cortical regions being sampled. In addition, breeds age at different rates. While this has been well documented in the musculoskeletal system, there are not yet enough data to determine whether the same applies to brain aging. To minimize the impact of these potential differences, we used each dog as its own control. This approach ensures that breed-related variability does not affect our findings.

Additionally, in the supplementary material we examined correlations between age, weight, and REM latency (a key variable) and found no significant associations (Supplementary Material S3). This suggests that, at least for the parameters analyzed here, body size was not a major determinant of the observed effects of trazodone.

Nonetheless, we acknowledge that broader physiological differences related to breed and size could play a role in sleep architecture in older or diseased populations. We have added this point to the limitations section of the manuscript and emphasized that future studies with larger and more homogeneous cohorts will be necessary to fully disentangle the contributions of breed, size, and age to trazodone’s effects on brain dynamics.

To include in the discusion manuscript:

*‘’Although our cohort included dogs of varying breeds and body sizes, which are known to influence longevity and certain physiological functions, the within-subject design of this study minimized these confounding effects. Moreover, no significant correlations were found between body weight, age, and key sleep parameters such as REM latency (*Supplementary Material S3 *). Nevertheless, we recognize that breed- and size-related factors may impact sleep and brain dynamics in broader populations, and future studies with larger, more homogeneous cohorts are warranted to address this question in depth.”*

2- How much different breeds skull size and structure affect to your analyses? With different sizes the power are also different? Especially since you use Wilcoxon test. How appropriate it would be to use paired methods where you actually compare same subject different statuses? I.e. how independent the samples are if the dogs are the same? Would paired analysis work? What are the shortcomings for using paired analysis in this study? Nevertheless, limitations section should be modified accordingly depending on which statistical analyses you use. Additionally, in your dataset is there any chance of multiple comparison problem that would affect the analyses?

As you can mention, Breed/skull size effects on EEG power. Skull thickness and geometry can attenuate and spatially filter scalp EEG, potentially affecting absolute power across dogs. We minimized this variance in three ways:

- **Within-subject design:** each dog was recorded with and without trazodone on separate days (counterbalanced order). Thus, all primary comparisons are intra-individual (same skull, montage, impedance, time-of-day), reducing breed/size confounds.
- **Standardized setup:** the same montage (F3/F4/Fz/Cz referenced to Oz), impedance threshold (<20 kΩ), and acquisition parameters were used in all sessions.
- **Robust metrics**: our primary measures (Lempel–Ziv complexity, permutation entropy, PLI/coherence) are amplitude-insensitive or less sensitive to uniform attenuation, further mitigating skull-related effects.

To further reduce skull- and size-related confounds, all PSD spectra shown in the manuscript are **relative PSD**, i.e., each spectrum was normalized by the total power in the 1–50 Hz range of the same animal. This normalization ensures that comparisons reflect differences in spectral distribution rather than absolute amplitude, providing a more accurate basis for between-condition contrasts. We clarify this explicitly in the revised Methods and Limitations sections.

Independence and statistical tests (paired vs. unpaired). Each dog contributed data in both conditions. We pre-aggregated five randomly selected, artifact-free epochs per state into a single representative value per dog. For states present in both conditions (Wakefulness, Drowsiness), paired Wilcoxon signed-rank tests were applied. For REM and NREM, however, not all trazodone sessions contained these states and two dogs lacked NREM entirely; thus, sample sizes differed and unpaired Wilcoxon rank-sum tests were required. Figures 3 and 5 were updated to reflect these revised statistics, and p-values for complexity and entropy measures are now provided in Supplementary Material S5.

Multiple comparisons. The only formal multiple-comparison setting was in the complexity/entropy analyses (four states within each group). Here, we used a Kruskal–Wallis global test followed by Dunn's post-hoc correction. Other analyses (PSD, connectivity, state transitions) involved trazodone vs. control comparisons within each state. While these analyses span multiple frequencies or electrode pairs, they were treated as exploratory; unadjusted p-values are reported transparently. We have clarified this in the revised Statistical Analysis and Limitations sections.

We also note that REM latency was not correlated with age or weight (Supplementary Material S3), further supporting that size differences did not bias key results.

We added in the text:

In Methods – Statistical Analysis:

“Data from both conditions were analyzed per dog, producing one representative value per state by averaging five randomly selected artifact-free epochs (N=1200 points/epoch). For Wakefulness and Drowsiness, we applied paired Wilcoxon signed-rank tests (two-sided). For REM and NREM, where some trazodone sessions lacked these states and two dogs lacked NREM, we used unpaired Wilcoxon rank-sum tests for state-wise comparisons. Normality was assessed with the Kolmogorov–Smirnov test. Multiple comparisons within the complexity/entropy analyses were controlled with Kruskal–Wallis tests followed by Dunn’s post-hoc correction.”

In Discussion – Limitations:

“Breed and skull size: Differences in skull thickness and head size can influence absolute EEG power via attenuation and smoothing. Our within-subject design, standardized montage/impedances, and use of amplitude-insensitive metrics (LZC, PE, PLI/coherence) reduce this confound. We additionally report relative band power to mitigate amplitude scaling. Statistics: Paired non-parametric tests were used for Wakefulness and Drowsiness, while unpaired tests were required for REM and NREM due to missing states. Paired designs assume symmetric distributions of differences and reduce usable sample size; these limitations are acknowledged.”

3- *I would presume that different dogs have different need for daytime naps. How this different need would affect to different sleep stages and results during daytime recording between 12.30PM and 1.30PM?*

We agree with the reviewer that daytime sleep propensity can vary across dogs depending on age, breed, body size, and individual differences. Prior studies have shown that total sleep time and sleep structure in dogs are influenced by these factors:

- **Age**: older dogs show more fragmented sleep and increased daytime napping (Takeuchi & Harada, 2002; Mondino et al., 2021).
- **Breed/size**: large-breed dogs tend to sleep longer overall, while small breeds often have shorter but more frequent naps (Zanghi et al., 2013).
- **Individual variability**: lifestyle and environment also affect daytime sleep patterns (Kis et al., 2014).

In our study, we minimized the potential confound by using a within-subject design, where each dog served as its own control under trazodone and non-trazodone conditions. This design helps

control for individual variability in daytime sleep need. Nevertheless, we acknowledge that differences in baseline sleep propensity between dogs may have increased inter-individual variability in the results. We have added this note to the Limitations section.

In the Supplementary Material S1, we provide the time spent in each sleep state and the latency to each state for every individual dog.

We added in the discusion manuscript

"Daytime sleep propensity varies between dogs depending on age, breed, and individual characteristics (Takeuchi & Harada, 2002; Zanghi et al., 2013; Kis et al., 2014; Mondino et al., 2021). Although our within-subject design minimized the impact of such variability on trazodone vs. control comparisons, it may have contributed to inter-individual variability in baseline measures."

- Takeuchi, T., & Harada, E. (2002). *Age-related changes in sleep–wake rhythm in dog*. Physiology & Behavior, 75(3), 217–221.
- Zanghi, B. M., Kerr, W., Araujo, J. A., Milgram, N. W. (2013). *Effect of age and feeding schedule on diurnal rest/activity rhythms in dogs*. Journal of Veterinary Behavior, 8(4), 195–203.
- Kis, A., Szakadát, S., Kovács, E., Gácsi, M., Simor, P., Gombos, F., Topál, J., & Bódizs, R. (2014). *Development of a non-invasive polysomnography technique for dogs (Canis familiaris)*. Physiology & Behavior, 130, 149–156.
- Mondino, A., Delucchi, L., Moeser, A., Cerda-Gonzalez, S., & Vanini, G. (2021). *Sleep disorders in dogs: a pathophysiological and clinical review*. Topics in Companion Animal Medicine, 43, 100516.

4- ***In your "Signal preprocessing" chapter, you say "Artifact-free, stable epochs were then carefully selected for quantitative EEG analysis." -> how many epochs were selected for each subject? I would be interested number of epoch or % share of whole recording that was analyzable. If there were some discrepancies between subject, e.g. for some had 90% good data while other had only 20%, especially if same subject % shares were totally different between two nights -> how you take these differences into account? Would not this affect your percentages of different sleep stages, if e.g. for some subject the artefact time happens to be e.g. during REM sleep? (It seems that you explain this later in line 150 forward, but still these numbers of epoch etc would be interesting to see?)***

We thank the reviewer for highlighting this important point. To address this, we quantified the number and proportion of artifact-free vs. noisy epochs for each sleep stage in both the control and trazodone conditions. As shown in Supplementary Material S1 Table 1 , which presents the mean ± SD of clean and noisy epochs and the percentage of usable data across all states, this analysis was performed across all dogs. For your information, we have also attached Supplementary Material S1 Table 2, which provides this data for each individual dog.

Based on these data, Wake and REM stages showed the greatest variability across dogs. For example, in the control condition, the mean proportion of artifact-free REM epochs was 54.95%

(±30.83%), while under trazodone it was 35.99% (±51.26%). Wake epochs were less abundant in absolute terms (mean 7.67% in control, 23.99% in trazodone). Drowsiness epochs also varied between dogs but remained relatively well-represented. In contrast, all NREM epochs were analyzable in both conditions because the dogs were in deep sleep and no movement was present.

To minimize the impact of these discrepancies on group analyses, we adopted two strategies:

1. **Uniform sampling across states:** For quantitative EEG metrics (PSD, LZC, PE, connectivity), we selected five random artifact-free epochs per state per dog (N = 1200 points/epoch). This ensured equal representation across conditions and states, independent of the total available data.
2. **Epoch-based scoring for sleep architecture:** For hypnogram analysis (time in states, transitions), all available epochs were included. This means the reported percentages represent the proportion of time across the entire recording, not just the artifact-free subsets. Importantly, artifact rejection was evenly distributed across states and was not systematically biased toward REM or any specific stage.

In summary, while variability in the proportion of usable data between dogs is inevitable in canine polysomnography due to movement and strong cranial musculature, our methods mitigate its impact on the analysis. We have now added these data and explanations to the Supplementary Material and have clarified this point in the Methods and Limitations sections.

To add in method section

''On average, the proportion of artifact-free epochs varied by sleep stage: under control conditions, 7.7% of Wake, 67.8% of Drowsiness, 54.9% of REM, and 100% of NREM epochs were usable. Administration of trazodone altered these proportions to 24.0%, 205.8%, 36.0%, and 100%, respectively (Supplementary Material S1 Table 1). To mitigate bias from these unequal artifact rates in quantitative EEG analysis, we uniformly sampled five artifact-free epochs per state per dog. For sleep architecture analysis (hypnogram scoring), all recorded epochs contributed to the state proportions, with artifact periods marked separately. The lack of systematic bias in artifact occurrence across stages ensured the representativeness of the calculated state percentages."

5- Sorry, I do not have access to your ref 21, and therefore I can't check on this myself, however it would be interesting to see (at least as in your answer not necessarily in manuscript) what are the 3 second epochs criteria for REM sleep and/or NREM also? If this would be possible? You say the usual guidelines in line 122-128, however this 3 second epochs puzzles me as I have got used to human AASM criteria with 30s epochs?

We thank the reviewer for this question. The convention of 30-second epochs derives from the AASM guidelines for human sleep scoring (Iber et al., 2007). However, in animal studies, much shorter epochs are typically used: for example, 2–10 seconds in cats and rodents (Cavelli et al., 2015; Torterolo et al., 2022). This shorter segmentation is advantageous for two reasons:

- **Artifact control.** Dogs were not sedated, and their strong cranial musculature produces frequent movement and muscle artifacts. Using shorter epochs makes it easier to identify and exclude clean segments.
- **Physiological resolution.** Transitions between wakefulness, drowsiness, NREM, and REM are often more rapid in animals than in humans. Shorter epochs allow for more precise identification of these transitions and of microarousals.

In canine sleep research, this approach is already validated: non-invasive polysomnography studies commonly use short epochs (Kis et al., 2014; Reicher et al., 2020). Our choice of 3-second epochs is therefore consistent with established practice in animal sleep studies and optimized for both data quality and physiological accuracy.

We added in the manuscrip method section

*"Sleep states were scored in **3-second epochs**, following established practice in canine and other animal polysomnography (Kis et al., 2014; Reicher et al., 2020; Cavelli et al., 2015; Torterolo et al., 2022). Short epochs were chosen for two reasons: (i) dogs were unsedated and often produced movement or muscle artifacts, which are easier to detect and exclude in shorter segments; and (ii) transitions between vigilance states occur more rapidly in dogs than in humans, so finer temporal resolution improves identification of NREM and REM states. Although human sleep scoring uses 30-second epochs (Iber et al., 2007), shorter epochs are the norm in animal sleep research.''*

– *I*ber, C., Ancoli-Israel, S., Chesson, A., & Quan, S. (2007). T*he AASM manual for the scoring of sleep and associated events*.
– Kis, A., Szakadát, S., Kovács, E., Gácsi, M., Simor, P., Gombos, F., Topál, J., & Bódizs, R. (2014). *Development of a non-invasive polysomnography technique for dogs (Canis familiaris)*. Physiology & Behavior, 130, 149–156.
– Reicher, V., Kis, A., Simor, P., Bódizs, R., & Gácsi, M. (2020). *Repeated afternoon sleep recordings indicate first-night-effect-like adaptation process in family dogs*. Journal of Sleep Research, 29(6), e13081.
– Cavelli, M., Castro-Zaballa, S., Mondino, A., Gonzalez, J., Rubido, N., Pascovich, C., & Torterolo, P. (2015). *Power and coherence analysis of the sleep EEG in the rat*. PLoS ONE, 10(5), e0126516.
– Torterolo, P., González, J., Castro-Zaballa, S., Cavelli, M., Mondino, A., Pascovich, C., Rubido, N., Murillo-Rodríguez, E., & Vanini, G. (2022). *Polysomnography in humans and animal models: basic procedures and analysis*. In Methodological Approaches for Sleep and Vigilance Research (pp. 17–32). Elsevier.

6- You probably mean Fig 2A in line 141 rather than Fig 1A?

Yes, you are all right , thank to point out

7- You also say in line 141 forward that "the hypnograms of animals with and without trazodone exhibit clear visual differences, indicating that trazodone affects sleep cycles, resulting in reduced variability." Yes the difference is clear, but I would not have raised that view up as for me it seems that during trazodone they actually sleep (NREM+REM) much less than in normal conditions (and yes the structure of sleep is totally different, but that is another topic)? Additionally, this is methods section so it seems odd to process results in this section, although it functions as bridge for LZC analysis. Maybe in that sense it is okay?
We introduced the analysis of hypnogram complexity because, despite only subtle changes in sleep states (Figure 2B), the overall hypnogram profiles differed markedly. To the best of our knowledge, this approach to analyzing hypnograms has not been applied before.

We rewrite the paragraph for better understanding.

First we remove the sentence pointed by you.

''In the second analysis, we examined the dynamics of sleep cycles by assessing the complexity of the hypnograms in both groups. ~~As shown in Figure 1A, the hypnograms of animals with and without trazodone exhibit clear visual differences, indicating that trazodone affects sleep cycles, resulting in reduced variability~~. To quantify this observation, we applied Lempel-Ziv complexity (LZC), a measure of sequence redundancy as detailed in the Method's in the following section.''

And in the second paragraph of the Lempel-Ziv subsection we added an improved introduction

''LZC was applied for the analysis of both hypnograms and EEG recordings''. For the hypnogram analysis, sleep states ...

8- ***Another point of those hypnograms, as I am not that familiar with studies done with dogs, is it normal to illustrate hypnogram from top to down as REM ->NREM -> drow ->wake? As usually hypnograms are illustrated top to down as wake -> REM -> N1 -> N2 -> N3? For me it seems odd to have wake section the lowest?***

We thank the reviewer for this observation. In fact, both formats for illustrating hypnograms are commonly used. Our group is more accustomed to using the format shown in Figure 2A.

9- ***In line 150 you actually explain my previous point regarding variability of number of epochs. However, you say that you five epochs? Meaning that you actually analyzed only 15 seconds? Each epoch had 1200 timepoints as usual for 400Hz resolution, however your bandpass was between 1-70Hz, so majority of this 400Hz goes unanalyzed. Is 15 seconds actually enough for these analyses? It seems quite low number? Additionally, if "epochs were chosen randomly to ensure fairness" how especially the connectivity analyses actually work with 3 second epochs?***

The number of artifact-free epochs varied widely between dogs and states. To ensure comparability across all dogs and all states, we selected the minimum number of epochs available for every dog in REM sleep (5 epochs). This allowed us to keep the dataset balanced across individuals and to avoid biases that would arise if some dogs contributed far more data than others.

We limited our analysis to 1–70 Hz because the key physiological changes of sleep architecture occur within the delta, theta, alpha, beta, and gamma ranges (<70 Hz). This is consistent with established human sleep scoring standards (Iber et al., 2007; Rechtschaffen & Kales, 1968) and canine polysomnography studies (Kis et al., 2014; Mondino et al., 2021; Torterolo et al., 2022). High-frequency activity above 70 Hz is less relevant to sleep physiology and more prone to noise and artifact.

Both PLI and coherence estimate phase and amplitude relationships between channels (Stam et al., 2007). These are computed across the time series and then averaged; thus, even in 3-second epochs we obtain hundreds to thousands of phase estimates. Both PLI and coherence compute instantaneous phase or amplitude relationships across time and then average them, which makes them robust even with short data segments. Indeed, previous work has shown that connectivity can be estimated reliably with windows as short as 1 second (Lachaux et al., 1999 ; Schoffelen & Gross, 2009). Thus, our use of 3-second non-overlapping epochs (5 per state per dog) falls well within established practice.

- Iber, C., Ancoli-Israel, S., Chesson, A., & Quan, S. (2007). *The AASM Manual for the Scoring of Sleep and Associated Events*.
- Rechtschaffen, A., & Kales, A. (1968). *A Manual of Standardized Terminology, Techniques and Scoring System for Sleep Stages of Human Subjects*.
- Kis, A., Szakadát, S., Kovács, E., Gácsi, M., Simor, P., Topál, J., & Bódizs, R. (2014). *Development of a non-invasive polysomnography technique for dogs (Canis familiaris)*. Physiology & Behavior, 130, 149–156.
- Mondino, A., Delucchi, L., Moeser, A., Cerda-Gonzalez, S., & Vanini, G. (2021). *Sleep disorders in dogs: a pathophysiological and clinical review*. Topics in Companion Animal Medicine, 43, 100516.
- Torterolo, P., Gonzalez, J., Castro-Zaballa, S., Cavelli, M., Mondino, A., Pascovich, C., Rubido, N., Murillo-Rodríguez, E., & Vanini, G. (2022). *Polysomnography in humans and animal models: basic procedures and analysis*. In Methodological Approaches for Sleep and Vigilance Research (pp. 17–32). Elsevier.

- Stam, C. J., Nolte, G., & Daffertshofer, A. (2007). *Phase lag index: assessment of functional connectivity from multichannel EEG and MEG with diminished bias from common sources*. Human Brain Mapping, 28(11), 1178–1193.
- Lachaux, J. P., Rodriguez, E., Martinerie, J., & Varela, F. J. (1999). *Measuring phase synchrony in brain signals*. Human Brain Mapping, 8(4), 194–208.
- Schoffelen, J. M., & Gross, J. (2009). *Source connectivity analysis with MEG and EEG*. Human Brain Mapping, 30(6), 1857–1865

10- In line 156 there is extra space before "Power" same thing with in line 212 before "Phase"

Thank you very much for this detail.

11- *In line 220 you use the most physiologically representative EEG bands, is this under normal conditions or also with trazodone? How much there are variability between subjects? Is it possible that something interesting is left out with the chosen bands in individual subjects?*

Although the boundaries of EEG frequency bands may vary slightly depending on the author and whether animal or human models are used, we adhered to the classical definitions. For the PLI analysis, which requires narrow frequency ranges, we selected representative narrow bands within these classical definitions. The same definitions were applied to both control and treated dogs.

12- In Fig 2B and line 261 onwards, how these deleted and not analyzed epochs affect these total time calculations?
- I would be interested to see similar charts for total analyzed time control vs trazodone and time wake vs sleep between two conditions. Not necessarily in actual manuscript but at least in your answers?

Thank you for this question. For the analyses presented in the section "Temporal duration and dynamics of sleep state cycles" (e.g., Fig. 2B), we used all epochs, including both artifact-free and noisy ones. This is because the aim of these analyses was to quantify the time spent in each behavioral state, rather than to analyze EEG signal quality. Artifact rejection was only applied in subsequent quantitative EEG analyses (PSD, entropy, connectivity).

Therefore, the total times reported in Fig. 2B and related text reflect the entire recording length (2 hours), with states scored continuously. This ensures that deleted epochs do not bias the percentage of time in Wake, Drowsiness, NREM, or REM.

13- Line 275 typo, "Thi" miss the S

Thank you very much for this detail.

14 - I am still thinking that your interpretation in line 277-278 is little off. Of course this might be the case, however, in my eyes the shown data does not fully support this. The thing that got me puzzled is mainly that trazodone should be "sleep medication" so why the dogs keep awake-drowsiness for longer time? And this affects naturally the whole structure of sleep in a timeframe of 120min?

Although trazodone produced only subtle effects on the total time spent in different behavioral states (Figure 2B), the hypnograms of control and treated animals differed markedly (Figure 2A), suggesting that trazodone altered the temporal evolution of sleep states. To quantify this difference, we applied Lempel–Ziv complexity (LZC) and transition probability analyses to the hypnograms, which confirmed the drug's effect (Figure 2D and 2E).

Regarding the hypnotic effects of trazodone, several important considerations should be taken into account. A well-recognized limitation of all FDA-approved hypnotic drugs is that they tend to alter normal nocturnal sleep architecture (see, for example, a review by one of our team members; Monti el al. 2016). Consequently, drug-induced sleep is not equivalent to physiological sleep and is likely less restorative. Another relevant issue is that trazodone, although it induces relaxation and facilitates sleep in dogs, is primarily classified as an antidepressant rather than an approved hypnotic. In the present study, we confirm that the sleep following trazodone administration is markedly different from physiological sleep.

– Monti, J. M., Torterolo, P., & Pandi-Perumal, S. R. (2016). *The effects of benzodiazepine and nonbenzodiazepine agents, ramelteon, low-dose doxepin, suvorexant, and selective serotonin 5-HT2A receptor antagonists and inverse agonists on sleep and wakefulness*. Clinical Medicine Insights: Therapeutics, 8, CMT-S38232.

15- In line 280 and Fig 2E you present transition probability between epochs? So, in this you use all epochs not only the 5 that you randomly selected before? With the used and analyzed data there seems to be some discrepancies in my eyes. Is it possible to explain these better? My confusion continues in PSD chapter as in lines 307-308 you say that number of samples in trazadone group is 60% less (which, as I have said before, would be interesting to see as in table or chart), but how this is possible if you have selected 5 epochs in each state for every subject as stated in the beginning of EEG analysis chapter? I am sorry if I am not able comprehend this fully, but for me this seems odd?

We thank the reviewer for pointing out this. Indeed, two different approaches were used depending on the analysis:

1. **Transition probabilities (Fig. 2E)**: For these analyses, we used all epochs (artifact-free and noisy) in order anlyse the complete hypnograms. This is necessary because the transition matrix requires continuity across the entire 2-hour recording, not just isolated clean segments.
2. **Quantitative EEG analyses (PSD, complexity, connectivity)**: Here, to avoid bias from unequal numbers of artifact-free epochs per dog and per state, we randomly use five clean epochs per state per dog and averaged them to produce a single representative value per animal. Group statistics were then performed on these animal-level values.
3. **REM sample size issue**: As noted, 60% of dogs under trazodone did not enter REM sleep at all. Consequently, for REM we had fewer animals contributing data compared to the control condition. In Fig. 3D (PSD) the curves represent mean ± SD across dogs that did reach REM in each condition. This explains why the sample size for trazodone REM is smaller. Because of this reduction, paired statistics cannot be applied to REM, and we clarify this explicitly in the revision.

We will add a clarifying statement in the Methods (EEG analysis) section to make the difference between "all epochs" (for hypnogram/transition analyses) and "5 epochs per state" (for quantitative EEG) more transparent.

To improve in the method section

"It is important to note that different subsets of epochs were used depending on the analysis. For hypnogram quantification and transition probability analyses, ***all epochs (including noisy ones)*** *were scored to preserve continuity across the recording. For quantitative EEG analyses (PSD, LZC, PE, connectivity), we randomly selected* ***five clean epochs per state per dog*** *to ensure equal representation and reduce bias from unequal numbers of artifact-free segments. For REM, only dogs that entered this state contributed to the analysis, resulting in fewer animals in the trazodone condition."*

16- ***Fig.3 legend between lines 308 and 308 there is a typos? "anlysis" and "de"***

Thank you very much for this details, The mistakes were corrected.

17- Statement in line 329 comes with surprise, I think these statements and results should be explained first? How this affects the whole analyses as different dogs are included in analyses and therefore size, skull etc affect the results?

The statement of line 329 is: "However, it is important to note that only 40% of the dogs in the trazodone group entered REM sleep". Our results clearly show that trazodone reduces the probability of entering REM sleep. In fact, 60% of the animals did not enter REM sleep during the

2-hour nap recording, while the remaining animals exhibited a marked increase in REM sleep latency, as illustrated in the representative hypnogram (Figure 2A). This finding is not unexpected, since antidepressants—including serotonin and norepinephrine reuptake inhibitors, norepinephrine reuptake inhibitors, monoamine oxidase inhibitors, selective serotonin reuptake inhibitors, and tricyclic antidepressants—are well known to suppress REM sleep generation, despite pharmacological differences among individual drugs. This suppression is linked to the fact that these compounds increase synaptic levels of monoamines (serotonin, norepinephrine, or both), which must decrease for REM sleep to be initiated. From a physiological perspective, REM sleep requires the inhibition of serotonergic neurons in the dorsal and median raphe nuclei, as well as noradrenergic neurons in the locus coeruleus, which function as REM sleep-OFF neurons (see review by Torterolo et al. 2019). These neurons are considered permissive for REM sleep generation; that is, their activity must be suppressed to allow the onset of REM sleep.

The Reviewer also asked: How this affects the whole analyses as different dogs are included in analyses and therefore size, skull etc affect the results? We expected that individual differences among the dogs could increase variability in the results. However, despite the heterogeneity of our sample, the findings remained consistent.

– Torterolo, P., Monti, J. M., & Vanini, G. (2019). *Neurochemistry and Pharmacology of Sleep*. The Behavioral, Molecular, Pharmacological, and Clinical Basis of the Sleep-Wake Cycle, 45–83. https://doi.org/10.1016/b978-0-12-816430-3.00004-x

18- Extra space in line 361 (see method)

Thank you very much for this details, The mistake was corrected.

19- Typo? In Fig.5 legend "---analysis for all PARE of electrodes"? Pair?

Thank you very much for this details, The mistake was corrected.

20- Lines 404-409 same text two times, i.e. "The administration of trazodone over the time has been demonstrated to enhance the quality of sleep in humans and rats through a reduction in the proportion of time spent in a Wakefulness state and an increase REM, NREM sleep [54–56]. The administration of trazodone over the time has been demonstrated to enhance the quality of sleep in humans and rats through a reduction in the proportion of time spent in a Wakefulness state and an increase REM, NREM sleep [54–56]."

Thank you for pointing that out. The repetitive sentence has been removed.
